# Resident and attending physician perception of maladaptive response to stress in residents

**DOI:** 10.3402/meo.v19.25041

**Published:** 2014-11-17

**Authors:** Lee Ann Riesenberg, Katherine Berg, Dale Berg, Charity J. Morgan, Joshua Davis, Robyn Davis, Arielle Schaeffer, Robert Hargraves, Brian W. Little

**Affiliations:** 1 Department of Anesthesiology, The University of Alabama at Birmingham, Birmingham, AL, USA; 2 Rector Clinical Skills Simulation Center, Sidney Kimmel Medical College, Thomas Jefferson University, Philadelphia, PA, USA; 3 Department of Biostatistics, University of Alabama at Birmingham, Birmingham, AL, USA; 4 Sidney Kimmel Medical College, Thomas Jefferson University, Philadelphia, PA, USA; 5 Tufts University School of Medicine, Boston, MA, USA; 6 Christiana Care Health System, Newark, DE, USA

**Keywords:** stress, stress management, residents, graduate medical education

## Abstract

**Background:**

Residency stress has been shown to interfere with resident well-being and patient safety. We developed a survey research study designed to explore factors that may affect perception of a maladaptive response to stress.

**Methods:**

A 16-item survey with 12 Likert-type perception items was designed to determine how often respondents agreed or disagreed with statements regarding the resident on the trigger tape. A total of 438 respondents from multiple institutions completed surveys.

**Results:**

Attending physicians were more likely than residents to agree that the resident on the trigger tape was impaired, *p*<0.0001; needed to seek professional counseling, *p*=0.0003; should be removed from the service, *p*=0.002; was not receiving adequate support from the attending physician, *p*=0.007; and was a risk to patient safety, *p*=0.02. Attending physicians were also less likely to agree that the resident was a good role model, *p*=0.001, and that the resident should be able to resolve these issues herself/himself, *p*<0.0001.

**Conclusion:**

Our data suggest that resident physicians may not be able to adequately detect maladaptive responses to stress and that attending physicians may be more adept at recognizing this problem. More innovative faculty and resident development workshops should be created to teach and encourage physicians to better observe and detect residents who are displaying maladaptive responses to stress.

Residency is a stressful time for the physician-in-training. Stress in residents has been shown to affect health care provision and even patient safety. Studies have shown that residents report becoming more cynical (61%) and less humanistic (23%) over the course of their residency (1) and 42–45% meet criteria for depression (2, 3). In a 2011 study of 16,192 internal medicine residents, 52% met at least one criterion for burnout (4), and a smaller study of all residency programs found a similar rate (50%) (5). A literature review of 51 studies published from 1974 to 2009 found that burnout rates in medical students ranged from 28 to 45% and in residents from 27 to 75% (6). Excessive stress in residency may also lead to certain ineffective coping mechanisms such as alcohol and substance abuse. In 2006, 0.9% of emergency medicine residents surveyed reported alcohol dependence or impairment and 12.6% reported increasing their alcohol consumption during their residency (7). Furthermore, anesthesiology department chairs reported a known drug abuse rate of 1.6% among their residents (8). In extreme cases, excess stress can lead to suicide; rates of which are higher in physicians compared to the general population, 41% higher in males and 127% higher in females (9). Finally, resident burnout, depression, and decreased quality of life have been shown to be associated with increased odds of a self-reported medical error (10).

Stress in residency training has received increasing attention from regulatory agencies. In July 2011, the Accreditation Council for Graduate Medical Education (ACGME) instituted Common Program Requirements that include new duty hour restrictions for residents (11). In addition, these requirements state that ‘[t]he program must be committed to and responsible for promoting patient safety and resident well-being in a supportive educational environment’ (11). Furthermore, they note that the institution and program director must ensure a culture of professionalism where ‘residents and faculty members must demonstrate an understanding and acceptance of their personal role in … [the] assurance of their fitness for duty … [and the] recognition of impairment, including illness and fatigue, in themselves and in their peers’ (11). Program directors, faculty members, and residents should be able to detect and provide appropriate interventions for residents who exhibit symptoms of a maladaptive response to stress.

To meet this need, we developed a 60- to 90-min workshop that included trigger tapes of a stressed resident, PowerPoint slides, discussion prompts, speaker notes, and resource handouts for participants. These resources are available on MedEdPORTAL (12). In addition, we developed a survey research study designed to explore factors that may affect perception of a maladaptive response to stress.

We had two hypotheses. First, that the gender of the resident on the trigger tape would affect survey responses. Specifically, that respondents would report more stress and impairment when the same behaviors where portrayed by a female resident. Second, we hypothesized that resident and attending physician responses would differ, with attending physicians reporting more stress and impairment.

## Methods

The research design was a cross-sectional survey study. The study was reviewed by the Institutional Review Boards (IRBs) at both institutions (Thomas Jefferson University, Philadelphia, Pennsylvania, and Christiana Care Health System, Newark, Delaware) and granted exempt status.

### Workshop

We developed a 60- to 90-min workshop for faculty, residents, and other health care professionals that focused on identifying stressed residents and ways to help a resident manifesting a maladaptive response to stress. The workshop included trigger tapes, a PowerPoint presentation with presenter notes, and participant resource handouts. This workshop was developed by an interdisciplinary, inter-institutional team from Thomas Jefferson University, an academic medical center in Philadelphia, Pennsylvania, and Christiana Care Health System, an independent academic medical center in northern Delaware. The workshop format and educational resources are available on MedEdPORTAL (12).

### Video trigger tapes

We recorded two versions of the trigger tapes, which were identical except for the sex of the actors. One version of the trigger tapes included a male resident and female intern and a second version included a female resident and male intern. Both versions used identical scripts. Professional actors were trained to follow the script exactly and finished trigger tapes were checked by four of the authors (LR, KB, DB, RH) to ensure identical portrayals.

Both versions of the trigger tapes consisted of two scenes demonstrating progressive maladaptive responses to stress. The first scene was designed to portray a resident manifesting some maladaptive signs of stress, including being abrupt with a colleague, irritable, and having poor eye contact when communicating. The second scene, involving the same resident and intern, was designed to portray a resident manifesting more severe maladaptive manifestations of stress, including anger, defensiveness, and discussing the need for alcohol consumption to get to sleep and caffeine pills to stay awake.

### Survey instrument

Our research team developed a 16-item survey instrument (Table 1). A preliminary survey was developed by two authors (DB and KB). The survey was pilot-tested for content validity, structure, and clarity among the other co-authors. The final version was designed to assess participant perception of a maladaptive response to stress in the resident in the trigger tapes.

**Table 1:** Stressed resident trigger tape survey designed to collect basic demographic information and respondent perception of the viewed resident's response to stress Please check the box that indicates how much you agree or disagree with the statements about Dr. Brown, the resident in the video clip(s).

	Strongly disagree	Disagree	Neither agree nor disagree	Agree	Strongly agree
1. The resident on the clip is under a great deal of stress.	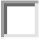	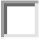	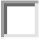	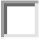	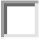
2. The resident on the clip is impaired.	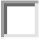	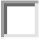	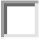	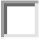	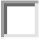
3. The resident on the clip needs to seek professional counseling.	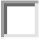	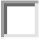	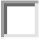	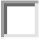	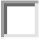
4. The problems the resident has are primarily related to at home issues.	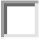	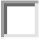	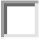	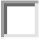	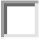
5. The resident in the clip should be removed from the service.	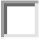	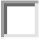	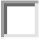	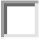	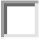
6. The resident on the clip is not receiving adequate support from the residency program.	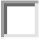	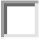	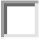	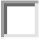	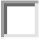
7. The resident on the clip is not receiving adequate support from the attending physician.	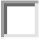	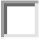	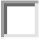	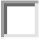	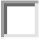
8. The resident on the clip has a substance abuse problem.	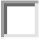	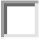	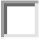	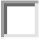	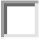
9. The resident on the clip is a risk to patient safety.	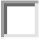	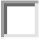	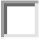	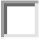	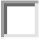
10. The resident on this clip is a good role model.	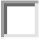	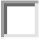	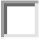	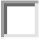	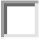
11. The resident on this clip demonstrates many of the common stressors that residents face today.	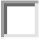	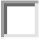	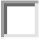	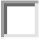	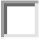
12. The resident on this clip should be able to resolve these issues her/himself.	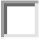	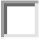	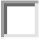	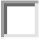	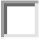
13. Gender 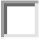 Male 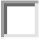 Female					
14. What is your health care profession? 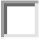 Physician 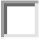 Resident, PGY ________ 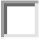 Nurse 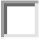 Nurse Practitioner 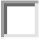 Physician Assistant 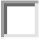 Other (please specify) _____________________________________					
15. How many years have you worked in health care? __________________					
16. What is your area of specialty? __________________________________					

After viewing both scenes of one version of the trigger tapes (male or female version), participants were asked to complete the survey. The first 12 items were statements that respondents were asked to respond to by indicating how much they agreed or disagreed with each item (using a five-point Likert scale, with responses ‘Strongly Disagree’, ‘Disagree’, ‘Neither Agree nor Disagree’, ‘Agree’, and ‘Strongly Agree’). In addition to assessing perception, the survey and trigger tapes were designed to explore factors that may affect perception of a maladaptive response to stress, such as gender of resident on the trigger tapes (male or female resident), gender of workshop participant, whether the participant was a resident or attending physician, number of years worked in health care, and area of specialty.

### Subjects

Data were collected from February 2009 to June 2011. This was a convenience sample of attending and resident physicians, medical students, and other health care professionals at local and regional workshops, grand rounds, and new resident orientation. Participants were asked to complete the survey after viewing both scenes from one version of the trigger tapes (male or female resident version). The faculty presenters shared with participants that completing the survey was voluntary. Participants were asked to place completed surveys in a specific location at the back of the room as they left the session and faculty presenters were careful to focus their attention on clean-up to reinforce the voluntary nature of this research.

Randomization was not possible, as each group had to view just one version of the two trigger tapes (male or female resident version). The use of each trigger tape version (male or female) was alternated sequentially as the workshop was presented to a different audience.

### Statistical analysis

For each of the 12 perception survey items, we fit ordinal logistic regression models to the responses, as the data arose from a five-point Likert scale. Multivariate analysis of variance assumes normal distribution of data; thus, it was determined not to be appropriate for this study. The covariates included were tape version (male or female), respondent gender, and respondent category (resident or attending physician). Workshop group also was included as a random effect in order to control for any possible correlation within groups. Only those respondents who described themselves as an attending (*n*=117) or resident (*n*=267) physician were included in the regression analysis. These two groups represented the majority (87.7%) of respondents.

## Results

The workshop was presented to 15 different groups, with a total of 438 respondents. Total attendance at each workshop was not recorded. However, faculty believed, based on completed survey counts after each session, that 80–90% of participants completed surveys. Two hundred and forty-one respondents (55%) watched the trigger tape version with a male resident and female intern, and 197 respondents (45%) watched the version with a female resident and male intern. Two hundred and twenty-four men and 207 women completed the survey; seven respondents did not specify their gender. The distribution of health care professions among the respondents is as follows: attending physician (*n*=117), resident physician (*n*=267), nurse (*n*=3), nurse practitioner (*n*=1), physician assistant (*n*=2), and other (*n*=44). Four respondents did not respond to this question. Three hundred and forty-one (77.9%) respondents listed the number of years that she/he had worked in health care; for these respondents, the average (range) was 8.96 (1–38) years.

The rates at which attending and resident physicians responded to the 12 survey items with either ‘Agree’ or ‘Strongly Agree’ as well as estimated odds ratios (OR) are displayed in Table 2. Attending physicians were statistically significantly more likely than residents to agree with five items: 2) ‘The resident on the clip is impaired’, 3) ‘The resident on the clip needs to seek professional counseling’, 5) ‘The resident in the clip should be removed from the service’, 7) ‘The resident on the clip is not receiving adequate support from the attending physician’, and 9) ‘The resident on the clip is a risk to patient safety’. Resident physicians were statistically significantly more likely than attending physicians to agree with two items: 10) ‘The resident on this clip is a good role model’, and 12) ‘The resident on this clip should be able to resolve these issues her/himself’. There were no statistically significant differences between the ratings of attending and resident physicians on the remaining five items.

**Table 2:** Percentage of respondents who gave the ratings ‘Agree’ or ‘Strongly Agree’, for attending and resident physicians after viewing trigger tapes at a workshop on stress in residency

	Attending physician (*n*=117) (%)	Resident physician (*n*=267) (%)	Odds ratio	95% Confidence interval	*p*
1. The resident on the clip is under a great deal of stress.	99.1	97.0	–	–	NS
2. The resident on the clip is impaired.	83.8	63.6	0.29	(0.18, 0.47)	<0.0001
3. The resident on the clip needs to seek professional counseling.	86.1	64.8	0.30	(0.18, 0.52)	0.0003
4. The problems the resident has are primarily related to at home issues.	16.5	19.9	–	–	NS
5. The resident in the clip should be removed from the service.	27.0	15.0	0.46	(0.28, 0.72)	0.002
6. The resident on the clip is not receiving adequate support from the residency program.	65.0	65.0	–	–	NS
7. The resident on the clip is not receiving adequate support from the attending physician.	87.2	75.1	0.44	(0.25, 0.77)	0.007
8. The resident on the clip has a substance abuse problem.	81.9	66.7	–	–	NS
9. The resident on the clip is a risk to patient safety.	87.8	72.9	0.52	(0.30, 0.91)	0.02
10. The resident on this clip is a good role model.	0.9	4.9	3.09	(1.68, 5.68)	0.001
11. The resident on this clip demonstrates many of the common stressors that residents face today.	93.2	94.7	–	–	NS
12. The resident on this clip should be able to resolve these issues herself/himself.	6.0	13.9	5.03	(2.82, 8.97)	<0.0001

Men were statistically significantly more likely than women to agree with two items: 4) ‘The problems the resident has are primarily related to at home issues’ (20.8% of men vs. 15.5% of women; OR=1.57, *p*=0.04, 95% confidence interval 1.02 to 2.38) and 12) ‘The resident on this clip should be able to resolve these issues her/himself’ (13.8% of men vs. 7.7% of women; OR=1.80, *p*=0.01, 95% confidence interval 1.02 to 2.75). There were no statistically significant differences between the ratings of men and women for the remaining items. Gender of the resident on the trigger tapes did not significantly contribute to the level of agreement on any of the 12 perception items.

## Discussion

Our interdisciplinary, inter-institutional team developed a stressed resident workshop that was presented at local and regional workshops, grand rounds, and new resident orientation. Workshop evaluation data (not presented here) were uniformly positive. In our study, both attending (99.1%) and resident (97%) physician respondents overwhelmingly agreed that the resident on the trigger tape (whether watching the male or female resident version) was under a great deal of stress.

We were surprised by the lack of statistically significant differences when viewers watched the male vs. female resident trigger tape version. We developed two versions of the trigger tapes, as our a priori hypothesis was that the gender of the resident on the trigger tapes would affect participant perceptions of stress and impairment on our survey.

However, when we compared male to female respondent answers, we did find two statistically significant differences. Male respondents agreed more frequently that the resident's problems were related to issues at home (*p*=0.04) and that the resident should be able to resolve his/her issues without outside help (*p*=0.01). While these two items were statistically significant, the percent agreement differences were small, with only 5–6% separating male from female survey responses. Given these small percentage differences, we believe it is premature to make any broad statements about the finding. These data merit further research and study.

As hypothesized, we did find statistically significant differences between resident and attending physician responses that do merit further discussion. Attending physicians were more likely than residents to identify the resident in the trigger tape as impaired, needing professional counseling, and presenting a risk to patient safety. Attending physicians also agreed more frequently that the resident should be removed from service and that s/he was not receiving adequate support from the attending.

In contrast, the residents were more likely to endorse the statements that the resident on the trigger tape (a) was a good role model and (b) should be able to resolve his/her issues without outside help. It should be noted that while statistically significant, resident endorsement of the preceding two items may not be clinically significant, as the relative agreement rates were low for both groups (0.9% vs. 4.9% and 6% vs. 13.9%, attending and resident physician, respectively). Having acknowledged this caveat, the overall results support the same conclusions.

Previous studies have noted that both resident and attending physicians consistently fail to recognize signs of stress, depression, substance abuse, and suicide in themselves and in others (13–15). As hypothesized, resident and attending physician responses differed, with attending physicians reporting more stress and impairment in the resident on the trigger tapes.

We believe that the lower percentage of resident physician respondents who perceived stress signs in the actors on the trigger tapes compared to attending physician respondents may result from resident respondents being around similar stress-related environments. This may cause them to accept a higher level of maladaptive stress behaviors or to be less sensitive to these responses to stress than attending physicians. In addition, if resident respondents are like the actors portraying residents on the trigger tapes, they may be in denial and thus not want to believe that the characteristics shown in the trigger tape correlate to a maladaptive response to stress. Finally, attending physicians may have been exposed previously to presentations on stress and maladaptive responses, which should result in their enhanced awareness of the problem.

Given that the resident respondents were less likely to identify maladaptive stress responses in the trigger tapes than the attending physician respondents, the burden of stress detection on residents, may be overemphasized. Resident physicians may not adequately detect maladaptive responses to stress in themselves or in colleagues. In addition, Hochberg and colleagues (13) demonstrated that using an educational intervention improved resident physicians’ ability to learn the signs and symptoms of stress and that they retained this knowledge over time. These results support the need for continued efforts to teach residents how to recognize signs of stress, recognize maladaptive responses to stress, and develop more appropriate coping mechanisms.

This should not diminish the attending physician's responsibility to identify the signs of a maladaptive response to stress in residents. In addition, our study used self-report data, which may not translate into appropriate actions taken by attending physicians when they perceive maladaptive responses to stress in residents.

If resident and attending physicians are better able to recognize the signs of stress in residents and potentially intervene, then resident burnout may be diminished, which may lead to improved patient safety. Our findings reinforce the ACGME requirements that attending physicians must pay close attention to their residents and recognize illness, stress, and fatigue, in both themselves and their residents. Based on our results, we would encourage the development and implementation of faculty and resident development programs, similar to the workshop we developed, to address this need and to help teach resident and attending physicians about how to better observe and detect maladaptive responses to stress in residents.

Limitations of this study include not taking into account participant cultural or ethnic differences in responses to stress. Subspecialty differences are not reported, as 16 distinctly different specialties were listed by respondents and the number of many of these was too small to analyze. The data from this study may not be generalizable, as it was a convenience sample. Workshop attendance and participation in the survey was not required; therefore, there may be selection bias of respondents.

All participants were attending a workshop entitled ‘Early Detection and Intervention for the Stressed Resident’. The title and purpose of the workshop likely resulted in a response bias that favored identifying a maladaptive response to stress in the resident on the trigger tapes. In addition, surveys were collected after an introduction to the magnitude of the problem (i.e., statistics on burnout, alcohol abuse, depression, suicide, and the effect on empathy and patient safety) and after viewing both scenes of one of the trigger tapes (male or female version).

The workshop title and content of the introduction could have primed participants to look for signs of maladaptive response to stress in the trigger tape and subsequently agree that the resident was under a great deal of stress. This also may have resulted in respondents being more likely to respond in a similar fashion despite the version being viewed (male or female). As a result, the study may not have been designed well enough to detect differential attitudes toward residents of different gender when exhibiting maladaptive responses to stress. However, this would not explain the difference we found between resident and attending physician responses.

## Conclusion

We present data that suggest that resident physicians currently may not be able to adequately detect maladaptive responses to stress in themselves or in colleagues and that attending physicians may be more adept at recognizing this problem. We believe that resident and faculty development workshops should be created to teach and encourage resident and attending physicians to better observe and detect residents who are displaying maladaptive responses to stress.
